# Phenotypic Diversity in Domesticated and Wild Timothy Grass, and Closely Related Species for Forage Breeding

**DOI:** 10.3390/plants12193494

**Published:** 2023-10-07

**Authors:** Yousef Rahimi, Girma Bedada, Silvana Moreno, Anne-Maj Gustavsson, Pär K. Ingvarsson, Anna Westerbergh

**Affiliations:** 1 Linnean Centre for Plant Biology, Department of Plant Biology, BioCenter, Swedish University of Agricultural Sciences, 750 07 Uppsala, Sweden; girma.bedada@slu.se (G.B.); silvana.moreno@slu.se (S.M.); par.ingvarsson@slu.se (P.K.I.); anna.westerbergh@slu.se (A.W.); 2 Department of Crop Production Ecology, Swedish University of Agricultural Sciences, 901 83 Umeå, Sweden; anne-maj.gustavsson@slu.se

**Keywords:** biomass, development, forage crop, genetic resources, perennial, *Phleum alpinum*, *Phleum nodosum*, *Phleum pratense*, wild relatives

## Abstract

Timothy grass (*Phleum pratense* L.) is one of the most important forage crops in temperate regions. Forage production, however, faces many challenges, and new cultivars adapted to a changing climate are needed. Wild populations and relatives of timothy may serve as valuable genetic resources in the breeding of improved cultivars. The aim of our study is to provide knowledge about the phenotypic diversity in domesticated (cultivars, breeding lines and landraces) and wild timothy and two closely related species, *P. nodosum* (lowland species) and *P. alpinum*, (high altitude species) to identify potential genetic resources. A total of 244 accessions of timothy and the two related species were studied for growth (plant height, fresh and dry weight) and plant development (days to stem elongation, days to booting and days to heading) in the field and in a greenhouse. We found a large diversity in development and growth between the three *Phleum* species, as well as between the accessions within each species. Timothy showed the highest growth, but no significant difference was found between wild accessions and cultivars of timothy in fresh and dry weight. However, these two groups of accessions showed significant differences in plant development, where timothy cultivars as a group reached flowering earlier than the wild accessions. This suggests that there has not been a strong directional selection towards increased yield during the domestication and breeding of timothy; rather, timothy has been changed for other traits such as earlier heading. Principal component analysis and cluster analysis based on all traits revealed distinct clusters. Accessions falling within the same cluster showed similarities in the development and growth rather than the type of accession. The large diversity found in this study shows the potential of using timothy accessions as genetic resources in crosses with existing cultivars. Also, accessions of *P. nodosum* with favorable traits can be candidates for the domestication of a novel forage crop, and the high-altitude relative *P. alpinum* may be a source of genes for the development of more cold and stresstolerant cultivars.

## 1. Introduction

Timothy (*Phleum pratense* L. subsp. *pratense*) is one of the most important forage crops in temperate regions. It is an outcrossing, short-lived perennial with shallow roots and, due to its winter hardiness, it is the preferred species for forage grass cultivation at higher latitudes in Northern Europe, East Asia and North America [1]. Timothy is a leafy and tall-growing grass with high biomass production, as well as high nutritive value and digestibility compared to many other forage grasses [2]. The forage quality of timothy is regulated by the developmental stage of the plant and is usually highest during the early growth of the crop when most of the tillers are in the vegetative stage [3,4].

Biomass production increases with age when the tillers elongate and reach the flowering stage. In contrast to some other forage crops such as *Lolium perenne* L. and *Festuca arundinacea* Schreb., timothy can form elongated and heading tillers without vernalization (cold treatment) [5,6]. However, flowering is stimulated by vernalization, especially in northern accessions [6,7,8,9]. Flowering and stem elongation are also stimulated by a longer photoperiod [5,8,9,10].

New tillers are formed from buds in leaf sheaths at the base of the stem. The stem bases (corms) are swollen and can serve as energy reserves [11]. With its rapid growth and formation of tillers, timothy can be harvested two to four times per season. However, the buds and corms in the crown are sensitive to trampling and grazing by cattle, which decreases the production of tillers. Another limitation for its persistence in pure stands or in mixtures with perennial legumes and other forage grasses is its shallow root system and its sensitivity to drought [12].

Timothy, together with fourteen perennial and annual species as well as several subspecies, form the genus *Phleum* [13,14]. Besides timothy, several other of these species are domesticated and cultivated as forage crops. The *Phleum* species vary in ploidy level from diploid to octoploid [13,14]. Even different ploidy forms are described within the same taxa. The polyploid forms are presumed to be the result of hybridization events and subsequent polyploidizations. The most common form of timothy is hexaploid, with 42 chromosomes (2n = 6x = 42). Hexaploid timothy is native to Europe, except for the Mediterranean areas, and the cultivated crop was domesticated from hexaploid wild populations in Northern Europe. Wild timothy populations grow in lowland areas throughout Europe.

The genetic relationship between the *Phleum* species and the origin of hexaploid timothy have puzzled scientists over the years and are still not fully understood. Based on genetic analysis, the similarity between hexaploid timothy and the diploid relative *P. nodosum* (syn. *P. pratense* L. subsp. *bertolonii (DC.) Bornm.* and *P. bertolonii (DC.) Bornm.*, 2n = 2x = 14) was found in chloroplast trnL intron DNA sequences [14]. Genetic similarities were also found between hexaploid timothy and the diploid *P. alpinum* subsp. *rhaeticum* Humphries (2n = 2x = 14). A hybridization is suggested to have occurred between the two diploid species *P. nodosum* and *P. alpinum* subsp. *rhaeticum* in the Italian Alps, which resulted in an allotetraploid *Phleum*. Moreover, cytological studies suggest the presence of two genomes of *P. nodosum* in hexaploid timothy [15,16]. Thus, the allotetraploid may have hybridized with northern European populations of *P. nodosum*, and with subsequent polyploidization, formed the hexaploid timothy, *P. pratense* subsp. *pratense*.

Wild populations of the different *Phleum* species originate from various geographical regions and are adapted to different habitats such as meadows and grasslands. Hybridization and polyploidy events in the evolution of *Phleum* are likely to have played an important role in the geographical and ecological patterns as well as in the diversification of the species. Also, variation in the direction and intensity of natural selection may have resulted in locally adapted populations within the species. Due to the strong human selection during crop domestication, the species may have undergone large bottlenecks, leaving much of the traits and genes behind in the wild populations [17,18,19]. These traits may be of large interest for the improvement of crops [20]. The wild populations of timothy and the related *Phleum* species are therefore potential genetic resources for the development of new high-yielding timothy cultivars adapted to a changing climate.

In this study, we investigated the development and growth in domesticated and wild hexaploid timothy and the two closely related species, the diploid *P. nodosum* and the tetraploid *P. alpinum* (2n = 4x = 28), growing in the Nordic countries. Taxonomically, the tetraploid *P. alpinum* belongs to the same species as the diploid *P. alpinum* subsp. *rhaeticum* [13,14]. The wild populations of *P. alpinum* commonly grow on meadows, riverbanks, roadsides and in birch forests at high elevations. At northern latitudes *P. alpinum* is found in Iceland, Norway, northern Sweden and northern Finland. Wild populations of *P. nodosum* are commonly found on meadows at low elevations. At northern latitudes, it has a more southern distribution than *P. alpinum*. Wild populations of timothy are found throughout the Nordic countries and grow at low elevations on meadows and in human-impacted and disturbed soils. The wild and domesticated accessions were studied both in the greenhouse and in the field to address the following questions: Do the three *Phleum* species differ in growth and development? Does the pattern of phenotypic diversity vary within and between the *Phleum* species? Do the wild and domesticated accessions of timothy differ in growth and development? Do some accessions show traits of interest for future breeding, and are therefore important genetic resources?

## 2. Results

### 2.1. Survival in the Field

The survival after the first winter was high in all groups of timothy accessions comprising wild, semi-wild, landraces, breeding lines and cultivars (100% of the accessions and from 93% to 96% of the plants; Table 1). The survival was also high in the cultivars and in the wild accessions of *P. nodosum* (100% of the accessions and from 92% to 96% of the plants). However, in *P. alpinum,* only 67% of the wild accessions and 66% of the plants survived the first winter.

### 2.2. Phenotypic Differences between Species

The ANOVA results demonstrated significant differences between the three *Phleum* species for all the studied growth traits and developmental stages under both field and greenhouse conditions (Tables S1 and S2). In the field, we observed significant block effects but failed to detect such effects in the greenhouse experiment. *P. nodosum* and *P. alpinum* showed similar days to booting (DTB) and days to heading (DTH) in the field, while timothy reached booting and heading later (Tukey HSD, *p* < 0.05; Table S3). However, in the greenhouse, *P. alpinum* and timothy showed similar development and reached booting and heading later than *P. nodosum* (Tukey HSD, *p* < 0.05; Table S4). In the field, the highest fresh weight (FW), dry weight (DW) and plant height (PH) were found in timothy (Tukey HSD, *p* < 0.05; Table S3), while in the greenhouse *P. nodosum* had the highest means of FW, DW and PH (Tukey HSD, *p* < 0.05; Table S4).

### 2.3. Variation in Growth Traits among Accessions

#### 2.3.1. Field Trial

**Figure 1:**
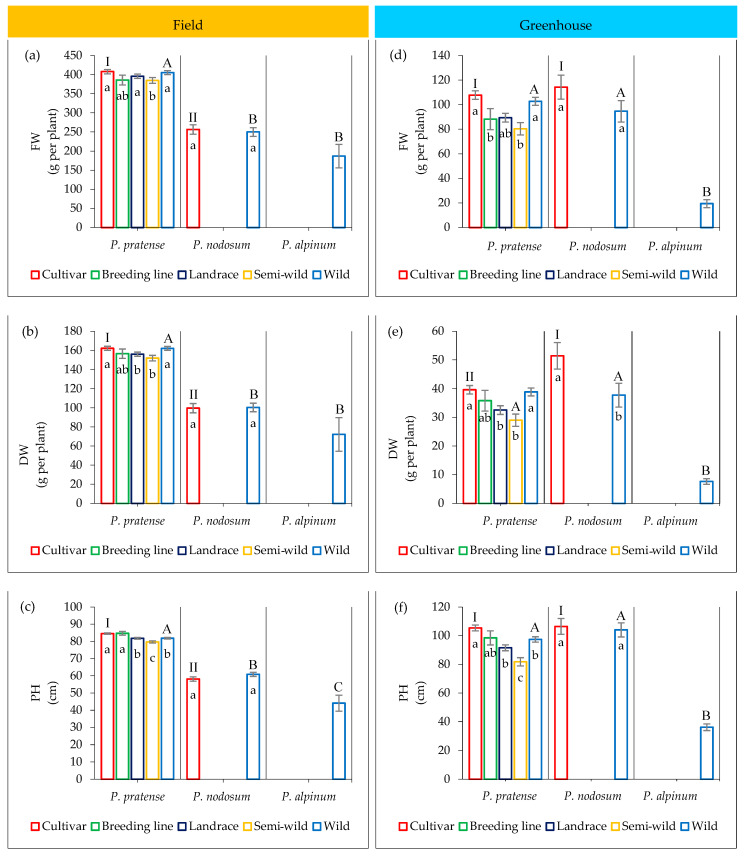
Mean and SD of fresh weight (FW), dry weight (DW) and plant height (PH) in different groups of accessions of *P. pratense*, *P. nodosum* and *P. alpinum* in the field (**a**–**c**) and in the greenhouse (**d**–**f**). Mean values that do not share the same letter are significantly different among accessions according to Student’s *t*-test, *p* < 0.05 for comparing two groups of accessions, and Tukey HSD, *p* < 0.05 for comparing more than two groups. Letters inside the bars show comparisons between groups of accessions within each species and capital letters outside the bars show comparisons between the groups of wild accessions of the three species, and Roman letters show comparisons between groups of cultivars of *P. pratense* and *P. nodosum*.

The mean FW and DW were about one-third higher in almost all groups of accessions of timothy compared to the cultivars and wild accessions of *P. nodosum,* and almost twice as high compared to the wild accessions of *P. alpinum* (Figure 1a,b). Interestingly, no significant difference was found between cultivars and wild accessions of timothy in FW or in DW (Tukey HSD, *p* > 0.05), while the semi-wild accessions had a significantly lower FW and DW. Similarly, no significant difference was observed between cultivars and wild accessions of *P. nodosum* (Student’s *t*-test, *p* > 0.05).

A similar pattern was also found for PH. All groups of timothy accessions showed about one-third higher mean than the *P. nodosum* cultivars and wild accessions, and more than twice the mean of *P. alpinum* (Figure 1c). There was no significant difference in PH between wild and cultivated accessions of *P. nodosum* (Student’s *t*-test, *p* > 0.05). However, within timothy the wild and semi-wild accessions as well as the landraces showed significantly lower PH than the breeding lines and cultivars (Tukey HSD, *p* < 0.05).

#### 2.3.2. Greenhouse Trial

The mean FW and DW of all groups of accessions in all species were significantly lower in the greenhouse than in the field (Figure 1d,e). For timothy, the FW and DW were about one-fourth of the yield in the field, and for *P. alpinum* they were only one-fifth of the yield in the field. However, in *P. nodosum* the yield was only reduced by half in the greenhouse compared to the field.

Even though the FW and DW were lower in the greenhouse, the pattern of variation among groups of timothy accessions was similar to the pattern found in the field, where no significant difference was found between the cultivars and the wild accessions (Tukey HSD, *p* > 0.05). In contrast to the field trial, the DW was significantly higher in the cultivars than in the wild accessions of *P. nodosum* (Student’s *t*-test, *p* < 0.05).

The mean PH was higher in the greenhouse than in the field for all groups of timothy accessions, except for the semi-wilds (Figure 1f), but the pattern of variation was similar to that found in the field.

### 2.4. Variation in Development among Accessions

#### 2.4.1. Field Trial

**Figure 2:**
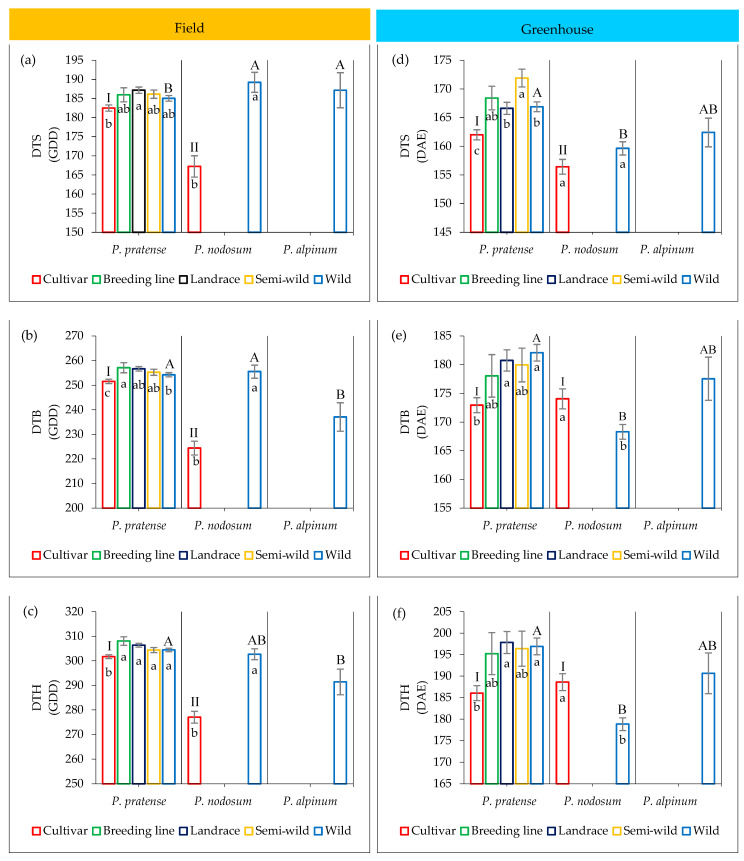
Mean and SD of days to stem elongation (DTS), days to booting (DTB) and days to heading (DTH) in different groups of accessions of *P. pratense*, *P. nodosum* and *P. alpinum* in the field based on growing degree days (GDD, **a**–**c**) and in the greenhouse based on days after emergence (DAE, **d**–**f**). Mean values that do not share the same letter are significantly different among accessions according to Student’s *t*-test, *p* < 0.05 for comparing two groups of accessions, and Tukey HSD, *p* < 0.05 for comparing more than two groups. Letters inside the bars show comparisons between groups of accessions within each species and capital letters outside the bars show comparisons between the groups of wild accessions of three species, and Roman letters show comparisons between groups of cultivars of *P. pratense* and *P. nodosum*.

A partially deviant pattern of variation was found when comparing the development within and between the three *Phleum* species compared to the pattern of variation for the growth traits. In timothy, the mean of DTB and DTH was significantly lower in the cultivars compared to the other groups of accessions, while for days to stem elongation (DTS) it was only significantly different from the landraces (Tukey HSD, *p* < 0.05; Figure 2a–c). In *P. nodosum*, the cultivars showed a significantly lower mean than the wild accessions for all developmental stages. In other words, the cultivars of both timothy and *P. nodosum* reached the three developmental stages earlier than their wild accessions did. Moreover, the *P. nodosum* cultivars reached these developmental stages earlier than the timothy cultivars. The wild accessions of timothy and *P. nodosum* did not differ significantly in DTB or DTH, while the wild accessions of *P. alpinum* reached booting earlier than timothy and *P. nodosum*, and reached heading earlier than the wild timothy accessions.

#### 2.4.2. Greenhouse Trial

In timothy, two wild accessions, four semi-wild accessions, four landraces, one breeding line and two cultivars only formed vegetative tillers at the end of the greenhouse trial about six months after the vernalization treatment. In addition, twelve wild accessions, nine semi-wild accessions, nine landraces, two breeding lines and four cultivars remained in the elongated stage at the end of the trial. For the plants that reached heading, the results from the greenhouse trial showed a pattern similar to what was found in the field for the developmental traits (Figure 2d,f), where cultivars had a significantly lower mean of DTS compared to the other groups of timothy accessions (Tukey HSD, *p* < 0.05). The cultivars also showed a significantly lower DTB and DTH compared to the landraces and the wild accessions of timothy (Tukey HSD, *p* < 0.05).

The pattern of variation was, however, different for *P. nodosum*. In contrast to the field trial, the cultivars reached booting and heading later than the wild accessions. Moreover, the development of the wild accessions of *P. alpinum* was rather different in the greenhouse than in the field since 12 out of the 18 accessions (86%) did not form elongated or heading tillers in the greenhouse.

### 2.5. Correlation between Traits

#### 2.5.1. Field Trial

**Figure 3:**
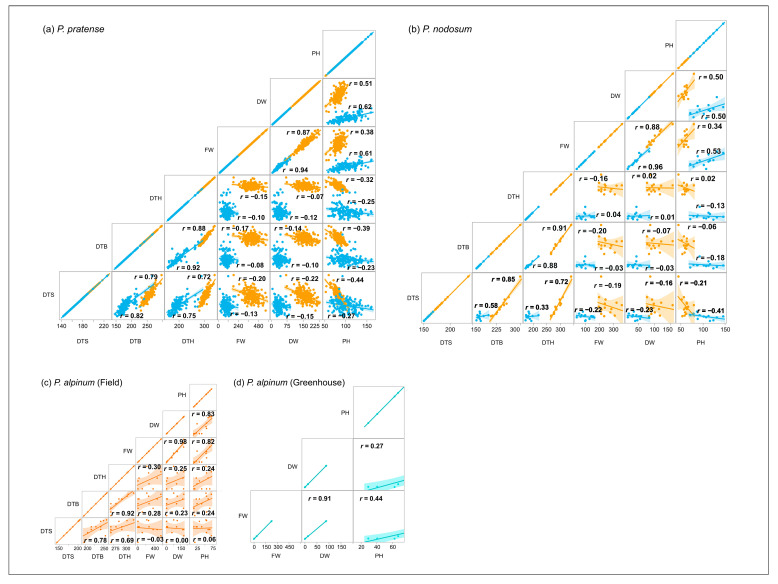
Correlation analysis between the studied traits, days to stem elongation (DTS), days to booting (DTB), days to heading (DTH), fresh weight (FW), dry weight (DW) and plant height (PH) in the field (brown) and in the greenhouse (blue). (**a**) *P. pratense*, (**b**) *P. nodosum* and (**c**) *P. alpinum* in the field and (**d**) *P. alpinum* in the greenhouse.

A strong positive correlation was found between the variation in all developmental stages (DTS, DTB and DTH) in all three species in the field (Figure 3a–c). FW and DW were also strongly correlated in all three species, while FW and PH, as well as DW and PH, showed a weaker correlation in both timothy and *P. nodosum*. The variation in the growth traits was not strongly correlated with the variation in DTS, DTB and DTH in all three species. In fact, we found no correlation between most of the growth and developmental traits in *P. nodosum* and *P. alpinum*. In timothy, no correlations or weak correlations were found between the two types of traits.

#### 2.5.2. Greenhouse Trial

Only the accessions that developed stem elongation, booting or heading in the greenhouse trial were included in the correlation studies. In timothy, a similar pattern of correlation between traits as seen in the field was also found in the greenhouse (Figure 3a). This was also true for *P. nodosum*, except for the weaker correlation between DTS and DTB, and between DTS and DTH, and the stronger correlation between FW and PH found in the greenhouse (Figure 3b). Moreover, *P. alpinum* plants showed a weaker correlation between FW and PH, and between DW and PH in the greenhouse (Figure 3d). Due to the low number of *P. alpinum* plants that formed elongated and flowering tillers, we did not perform a correlation test between the three developmental stages or between them and the growth traits.

### 2.6. Patterns of Phenotypic Diversity

#### 2.6.1. Field Trial

**Figure 4:**
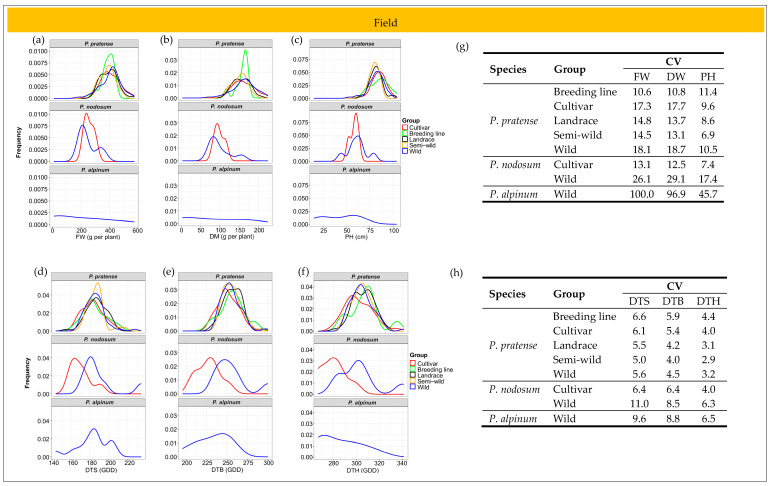
Distribution of studied traits in different groups of accessions of *P. pratense*, *P. nodosum* and *P. alpinum* in the field. (**a**) Fresh weight (FW), (**b**) dry weight (DW), (**c**) plant height (PH), (**d**) days to stem elongation (DTS), (**e**) days to booting (DTB), (**f**) days to heading (DTH), (**g**) coefficient of variation (CV) of growth traits, (**h**) CV of developmental stages.

The distribution of the studied traits varied between species and between groups of accessions within the species (Figure 4a–f). Coefficient of variation (CV) was calculated for each trait to evaluate the phenotypic diversity among the species and groups of accessions. *P. alpinum* showed a much higher CV than timothy and *P. nodosum* for all growth traits in the field (Figure 4g). In timothy, the wild accessions and cultivars showed a similar diversity and the highest CV for FW and DW, while the breeding lines had the highest CV for PH. However, in *P. nodosum*, the wild accessions had a much higher CV than the cultivars for all growth traits. The highest diversity in the developmental traits was found in *P. alpinum* and the wild accessions of *P. nodosum* (Figure 4h). In timothy, the cultivars and breeding lines showed larger diversity in DTS, DTB and DTH than the other groups of accessions.

**Figure 5:**
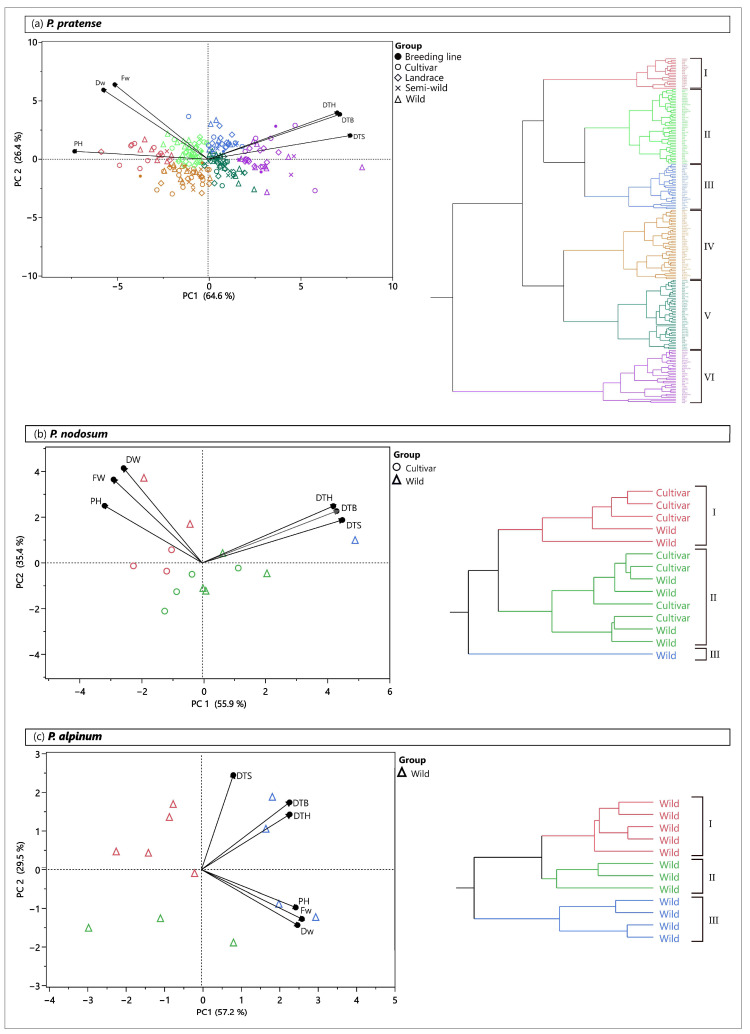
Principal component analysis (PCA) and cluster analysis based on all studied traits, days to stem elongation (DTS), days to booting (DTB), days to heading (DTH), fresh weight (FW), dry weight (DW) and plant height (PH) in the field for (**a**) *P. pratense*, (**b**) *P. nodosum* and (**c**) *P. alpinum*. Different colors in the PCA correspond to the clusters of accessions defined in the cluster analysis.

A principal component analysis (PCA) based on the variation in all growth traits and the developmental stages studied in the field showed a large phenotypic diversity among timothy accessions and formed six clusters (Figure 5a). In general, the accessions did not cluster according to the type of accessions (cultivar, breeding line, landrace, semi-wild and wild).

Timothy accessions falling within the same cluster in the PCA showed similarities in the development and growth traits rather than the type of accession. Accessions in cluster I (highlighted in red) had on average the highest PH, FW and DW, and reached the three developmental stages (DTS, DTB and DTH) earlier than the accessions in the other clusters (Figure 5a and Table 2). On the contrary, the accessions in cluster VI (highlighted in purple) showed on average the lowest yield and PH, and reached elongation, booting and heading later than any of the other clusters. The accessions in cluster II (highlighted in light green) and cluster III (highlighted in blue) had similar and relatively high FW and DW, but accessions in cluster II reached the three developmental stages earlier than those in cluster III. Both cluster IV (highlighted in brown) and cluster V (highlighted in dark green) consisted of relatively low-yielding accessions. The two clusters differed, however, in DTS, DTB and DTH. The total number of accessions of each group of accessions is shown in Table S5.

For *P. nodosum*, two clusters were formed with wild accessions and cultivars in both clusters (Figure 5b). In addition, a single wild accession did not cluster with any other accession. Cluster I (highlighted in red) consisted of accessions with, on average, the tallest plants as well as the highest FW and DW (Table 2). In contrast, the single accession (highlighted in blue) reached heading very late and had the lowest PH, FW and DW. The accessions in cluster II (highlighted in green) showed a phenotype in between the accessions in cluster I and the single accession.

The wild accessions of *P. alpinum* were divided into three clusters (Figure 5c). Cluster I (highlighted in red) was represented by accessions with very low PH, FW and DW, while cluster III (highlighted in blue) showed the highest yield (Table 2). Cluster II (highlighted in green) consisted of very early booting and heading accessions.

#### 2.6.2. Greenhouse Trial

**Figure 6:**
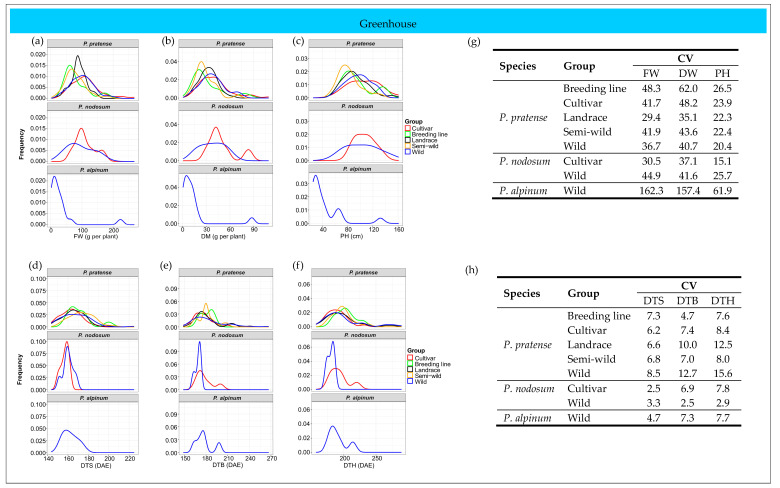
Distribution of studied traits in different groups of accessions of *P. pratense*, *P. nodosum* and *P. alpinum* in the greenhouse. (**a**) Fresh weight (FW), (**b**) dry weight (DW), (**c**) plant height (PH), (**d**) days to stem elongation (DTS), (**e**) days to booting (DTB), (**f**) days to heading (DTH), (**g**) coefficient of variation (CV) of growth traits, (**h**) CV of developmental stages.

Differences in the distribution of the growth traits and the developmental stages were also found between species and between groups of accessions within species in the greenhouse trial (Figure 6a–f). However, the distribution pattern in the greenhouse differed partly from the pattern found in the field. For example, the variation in the distribution of PH was larger between groups of timothy accessions in the greenhouse than in the field (Figure 4c and Figure 6c) and the variation in the developmental traits was larger between the wild accessions and the cultivars of *P. nodosum* in the field (Figure 4d–f and Figure 6d–f).

Similar to what was found in the field trial, *P. alpinum* showed a much higher diversity for the growth traits than the other two species in the greenhouse (Figure 6g). Also, the wild accessions of *P. nodosum* had a higher CV than the *P. nodosum* cultivars for FW, DW and PH. While the wild accessions and cultivars had the highest diversity for FW and DW in the field, the breeding lines of timothy showed the highest CV for the growth traits in the greenhouse. The highest diversity for the developmental traits was found in the wild accessions of timothy, while the wild accessions of *P. nodosum* showed the lowest CV for DTB and DTH (Figure 6h).

**Figure 7:**
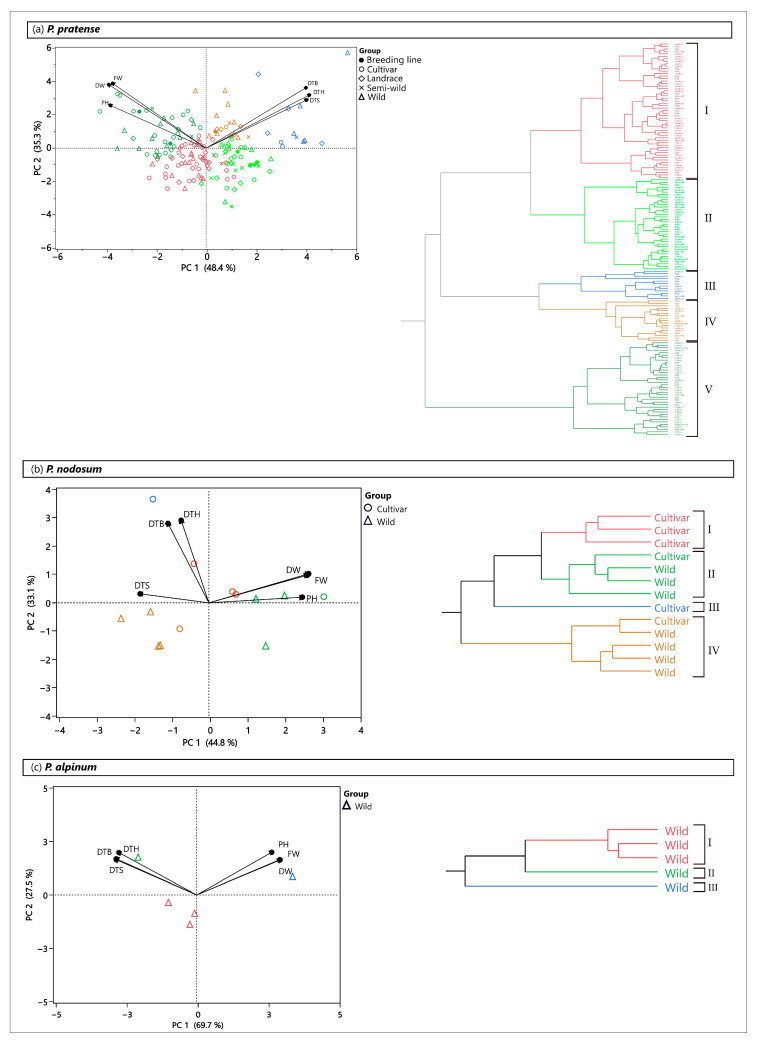
Principal component analysis (PCA) and cluster analysis based on all studied traits, days to stem elongation (DTS), days to booting (DTB), days to heading (DTH), fresh weight (FW), dry weight (DW) and plant height (PH) in the greenhouse for (**a**) *P. nodosum*, (**b**) *P. alpinum* and (**c**) *P. pratense*. Different colors in the PCA correspond to the clusters of accessions defined in the cluster analysis.

A PCA based on all studied traits in the greenhouse also showed a large diversity among accessions in all the three *Phleum* species (Figure 7). Among the timothy accessions that reached heading, five clusters were formed. Both domesticated and wild accessions were found in all clusters. Accessions in cluster V (highlighted in dark green) had the highest yield and PH and reached the three developmental stages early (Figure 7a and Table 3). Cluster II (highlighted in light green) and III (highlighted in blue) included both low-yielding accessions, but the accessions in cluster III reached heading much later than the accessions in cluster II. Mid-early accessions were found in cluster IV (highlighted in brown). These accessions showed high FW and DW. The total number of accessions of each group of accessions is shown in Table S6.

The accessions of *P. nodosum* were grouped into three clusters (one more cluster than in the field) and a single cultivar that did not cluster with any other accession (Figure 7b). Cluster I (highlighted in red), which comprised only cultivars, cluster II (highlighted in green) and cluster III (highlighted in blue) had all high and similar mean FW and DW, while cluster IV (highlighted in brown) showed a low yield (Table 3). All clusters reached stem elongation early, but differed in DTB and DTH, where accessions in cluster III were very late.

*P. alpinum* formed one cluster with three accessions (highlighted in red, Figure 7c). The other two accessions did not cluster with each other or any other *P. alpinum* accession. The mean FW and DW in the accessions were very low in cluster I and one of the single accessions (highlighted in green) compared to *P. nodosum* and timothy (Table 3). Cluster I and this single accession (highlighted in green) also showed late booting and heading, while the other single accession (highlighted in blue) was early and high yielding.

### 2.7. Heritability

The broad-sense heritability (*H*^2^) was lower in the field than in the greenhouse for all traits, except for PH which showed similar *H*^2^ in both trials (Table 4). The largest difference was found for the developmental stages where *H*^2^ was as high as 0.95 for both DTB and DTH in the greenhouse, while it was 0.37 for both traits in the field. In the greenhouse, the growth traits showed much lower *H*^2^ compared to the developmental stages. However, in the field, the heritability for the growth traits and the developmental stages was more similar.

## 3. Discussion

Agriculture is challenged by an increased demand for food for a growing human population and thereby an increased demand for animal feed during a changing climate. To respond to this challenge, high-yielding cultivars adapted to grow in a changing environment need to be developed. In this context, wild populations and closely related species to the crop may serve as valuable genetic resources for the development of improved cultivars. The aim of our study was to provide knowledge about the phenotypic diversity in the forage crop timothy and two of its closely related species, *P. nodosum* and *P. alpinum*. We studied different growth and plant developmental traits in domesticated and wild accessions and identified potential genetic resources for timothy breeding.

The three *Phleum* species showed variation in growth. All groups of timothy accessions had a significantly higher FW, DW and PH than *P. nodosum* and *P. alpinum* in the field. Moreover, *P. nodosum* formed taller tillers than *P. alpinum*. The greater growth of timothy was expected as it is one of the most important forage crops in temperate regions and cultivated for its high biomass production. Cultivars of *P. nodosum* and *P. alpinum* are developed, but to the best of our knowledge not used in mixtures with other forage crops. However, in the greenhouse *P. nodosum* showed similar growth to timothy, while the plants of *P. alpinum* were very small and most of them did not produce elongated tillers. The cultivation of plants under different growing conditions in the greenhouse compared to in the field, such as differences in temperature, photoperiod and water and nutrient content in the soil, most likely contributed to the overall lower FW and DW in the greenhouse. For example, a longer photoperiod has been shown to stimulate growth and biomass production in *P. alpinum* [21]. The variation in the growing conditions seems to have also affected the heritability of the studied traits in timothy, where the heritability was higher in the greenhouse than in the field for almost all traits.

*P. nodosum* was, however, less affected by the different growing conditions in the field and the greenhouse than timothy and *P. alpinum*. The adaptation to different habitats may explain the difference in performance among the species in the two trials. *P. nodosum* is characterized as a lowland species with rapid growth and spread, while *P. alpinum* is primarily found at higher altitudes adapted to harsh mountainous environments [22]. The diploid *P. nodosum* and the tetraploid *P. alpinum* carry different genomes [13,14]. The genomic form of *P. nodosum* is described as B_N_B_N_ and the genomic form of *P. alpinum* as R_E_R_E_XX. Our result suggests that the B_N_ genome carries genes for good and robust growth in different growing environments. In addition, cytological studies indicate that the hexaploid timothy carries two genomes of *P. nodosum* [15,16]. The polyploidization and duplication of the B_N_ genome in timothy is likely to have contributed to its large growth and biomass production. In fact, polyploidy has been shown to increase plant growth in wild plants and domesticated crops [23,24,25]. Polyploidization is an important tool in forage crop breeding, and the effect of genome duplication on biomass production and other traits would be of interest to explore.

A large phenotypic diversity was found within each *Phleum* species and the different groups of accessions. However, even though the diversity was high within the wild accessions and the cultivars of timothy, these two groups did not differ significantly in FW and DW in the field or in the greenhouse. Moreover, we did not find significant difference in spring growth between wild accessions and cultivars of *P. nodosum*. The similarity in the phenotype of wild accessions and cultivars as well as the large diversity among cultivars found in this study suggest that there has not been a strong directional selection towards increased yield during the domestication and breeding of timothy and *P. nodosum.* A strong directional selection towards a higher seed yield and other domestication traits such as resistance to seed shattering and apical dominance is found in annual grain crops [17,18,19,26], while in perennial forage crops, a high biomass production and forage quality are desirable [27,28,29,30]. The large changes in phenotypic traits in seed crops are a result of strong selection during many breeding cycles, while most of the forage crops have gone through relatively few cycles of selection. The human selection may also have been counteracted by cross-pollination in self-incompatible grasses such as timothy, increasing the diversity within the species.

In timothy, the undomesticated wild accessions showed a vigorous growth and high FW and DW, which are desirable traits for a forage crop. Our results suggest that the timothy cultivars have rather been selected and improved for other traits. We found a significant difference in plant development between the wild accessions and the cultivars of timothy both in the field and in the greenhouse. The group of timothy cultivars reached stem elongation, booting and heading earlier than the wild accessions. This shows that the breeding has favored a rapid plant development in spring growth, and thereby made repeated harvests of timothy possible during the same season. Also, in *P. nodosum,* the cultivars showed earlier stem elongation, booting and heading than the wild accessions in the field. The difference between the wild accessions and the cultivars in plant development was more pronounced in *P. nodosum* than in timothy, and in the field the *P. nodosum* cultivars reached heading much earlier than the timothy cultivars. The contrary was, however, found in the greenhouse, where the *P. nodosum* cultivars showed booting and heading later than the wild accessions, and reached heading at about the same time as the timothy cultivars. Differences in performance in the two growing environments were also found in timothy and *P. alpinum* accessions. Many domesticated and undomesticated accessions of timothy and a majority of the wild *P. alpinum* accessions did not flower in the greenhouse. The difference in the ability to flower in *P. alpinum* and timothy, and the opposite performance of the *P. nodosum* cultivars and wild accessions in the two growing environments, may be influenced by the difference in photoperiod. Also, the difference in length of vernalization between the six-week cold treatment in the greenhouse and the longer cold period in the field may have affected the development and ability to flower. Plants differ in their need for vernalization to flower, and winter-type plants of annual cereals and some perennial grasses such as ryegrass and tall fescue require vernalization [5,31,32].

The transition to the reproductive stage may also be affected by the photoperiod. In timothy, an increasing temperature and a longer photoperiod stimulate stem elongation during spring growth [5,8,9,10]. The need for vernalization for the transition to reproductive tillers differs between timothy accessions from different geographical origin [6,7,8,9]. While accessions in northern Scandinavia require vernalization to flower, more southern-growing accessions are not affected by the vernalization. This adaptation to the climate (e.g., photoperiod and temperature) at the cultivation and growing sites may explain the variation in heading time that we have found among accessions, and the different performance of some accessions in the field and the greenhouse. A weak correlation was found between the latitude of the geographical origin of the wild accessions of timothy and the heading time (Figure S1). A correlation between heading time and geographical origin in timothy has also been shown in other studies [7,10].

The large diversity that we have found in this study is of value for further pre-breeding studies. It also shows the potential to identify genetic resources for developing new improved timothy cultivars. The variation in days to stem elongation and flowering indicates the difference in response to photoperiod, where some accessions required a shorter photoperiod to reach flowering compared to other accessions. These accessions may therefore serve as important germplasm for development of improved cultivars for cultivation at lower latitudes. Accessions that respond to longer days for flowering could be used as germplasm for the development of new cultivars for northern latitudes. However, early cultivars having a vigorous growth and a high biomass production may have a lower nutritional value and forage quality than cultivars flowering later due to their faster production of elongated tillers [3,4]. This negative relationship between high yield and high forage quality is challenging in the breeding of forage grasses. Deviant accessions with high yield and delayed and suppressed flowering would therefore be of great interest for breeding. Interestingly, no strong correlation was found between the growth and plant developmental traits in our study, which indicates that these traits can be selected independently of each other. Timothy accessions with favorable traits could either be crossed with existing cultivars to transfer these traits into the crop or domesticated to make them adapted to grow on agricultural land.

Moreover, the relatively high yield of *P. nodosum* and its adaptation to low altitudes make this species an interesting candidate for the development of new forage crops. Selected accessions could be domesticated as a forage crop or crossed with already domesticated *P. nodosum* cultivars. On the other hand, *P. alpinum* may also be a potential source of genes for adaptation to colder and more stressful growing conditions. Genes from the wild relatives may also be transferred into the timothy forage crop through wide hybridization since Nordenskiöld [33] found that crosses between *P. nodosum* and *P. pratense*, and between *P. pratense* and *P. alpinum,* could give fertile hybrids.

## 4. Materials and Methods

### 4.1. Plant Material

**Figure 8:**
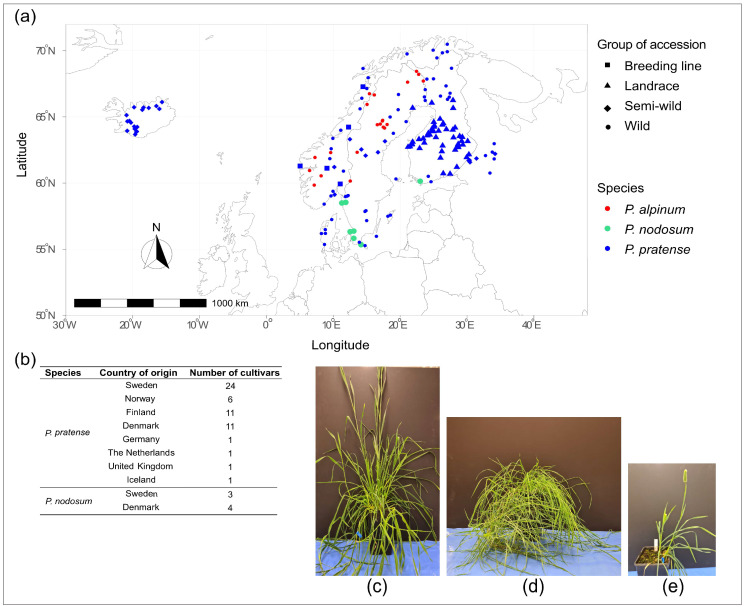
(**a**) Collection sites of wild and semi-wild accessions, landraces and breeding lines of *P. pratense*, *P. nodosum* and *P. alpinum*, and (**b**) country of origin of developed cultivars based on passport data from the genebank NordGen, Alnarp, Sweden. Two months old plants of (**c**) *P. pratense*, (**d**) *P. nodosum* and (**e**) *P. alpinum* grown in a greenhouse.

Clonal plants from 212 accessions of timothy (*P. pratense*), 14 accessions of *P. nodosum* and 18 accessions of *P. alpinum* originating from the Nordic countries, UK, Germany, the Netherlands and Russia were planted in the field (Table S7 and Figure 8). The same clonal plants, except four accessions (two timothy, one *P. nodosum* and one *P. alpinum*), were also studied in a greenhouse at the Plant Cultivation Facility, Uppsala BioCenter, Swedish University of Agricultural Sciences, Uppsala, Sweden. The seeds were provided by the genebank NordGen, Alnarp, Sweden. In the Nordic Baltic Genebanks Information System, *Phleum* accessions are described as wild, semi-wild (from populations nearby cultivated fields with potential gene flow between populations), landrace (from a cultivated variety adapted to a certain ecogeographical area), breeding line (group of related genotypes under evaluation in a breeding program) or cultivar (Table S7).

### 4.2. Pre-Cultivation and Cloning

Eight seeds of each accession were sown in low nutrient commercial potting soil (S-jord, Hasselfors Garden, Örebro, Sweden, Figure S2) in a climate chamber at the Plant Cultivation Facility, BioCenter, Swedish University of Agricultural Sciences, Uppsala, Sweden, with 16/8 h day/night photoperiod (350 µmol m^−2^ s^−1^ light), 22/17 °C day/night temperature and 65 percent humidity. After two months of growth, four randomly selected plants (genotypes) of each accession were cloned. Since the *Phleum* species are outcrossing, we considered each of the four plants within an accession genetically different. Vegetative tillers were gently separated from each other, and six of the tillers from each plant were individually transplanted into pots (15 cm × 15 cm × 20 cm) with commercial potting soil (P-jord, Hasselfors Garden, Örebro, Sweden). The cloned plants (totally six plants per genotype) were then cultivated in the greenhouse with a 16/8 h day/night photoperiod.

### 4.3. Field Trial

In July 2020, four plants of each genotype were transplanted in clay soil in a farmer’s field north of Uppsala, Central Sweden (60°00′ N, 17°42′ E). The field was located at an organic farm, and a low level of animal manure fertilizer was added. Manual weeding was carried out, and no herbicides were applied. In total, 244 *Phleum* accessions with 4 genotypes of each accession and 4 plants of each genotype were planted in a randomized complete block design with one plant of each genotype in each of the 4 blocks. The distance between the plants was 1 m within and between rows. The development and growth were studied during the season 2021, one year after planting, until the harvest in June the same year.

### 4.4. Greenhouse Trial

The other two plants of each genotype were grown in the greenhouse for two weeks and then given a vernalization treatment for six weeks at 4 °C under an 8 h photoperiod at 100 µmol m^−2^ s^−1^, to stimulate flowering. After the vernalization, the plants were transferred back to the greenhouse (16/8 h; 22/17 °C) and placed in two blocks with one replicate of each genotype randomized within each block. In total, 240 *Phleum* accessions with 4 genotypes of each accession and 2 plants of each genotype were evaluated from September 2020 until the last plant was harvested in March 2021. During the experiment, the plants were given nutrient solution (N: 102 mg/L (NH_4_: 40 mg/L, NO_3_: 62 mg/L), P: 20 mg/L, K: 86 mg/L, S: 8 mg/L, Ca: 6 mg/L, Mg: 8 mg/L, Fe: 0.34 mg/L, Mn: 0.4 mg/L, B: 0.2 mg/L, Zn: 0.06 mg/L, Cu: 0.03 mg/L, Mo: 0.08 mg/L; Wallco Miljöcenter AB, Arlöv, Sweden) twice a month.

### 4.5. Evaluated Traits

The clonal plants grown in the greenhouse and in the field were individually studied for the same growth traits and developmental stages (Table 5). Development was evaluated by recording the number of days to stem elongation (DTS), days to booting (DTB) and days to heading (DTH). We considered a plant to have reached stem elongation when the first tiller internode started to elongate, and the inflorescence was palpable at least 1 cm above the stem base [34] in about one-fourth of the total number of tillers (vegetative and elongated tillers). A plant was considered to have reached booting when the tip of the inflorescence was palpable in the flag leaf sheath and heading when the tip of the head was visible above the flag leaf in about one-fourth of the total number of tillers (vegetative, elongated and heading tillers). In the greenhouse, DTS, DTB and DTH were measured by recording the number of days after emergence of the coleoptile, including the time of vernalization.

In the field, the number of days to reach a specific developmental stage was recorded according to the day-of-year calendar. Based on that, DTS, DTB and DTH were calculated as accumulated growing degree days (*GDD*)

(1) $$GDD=\frac{T_{max}+T_{min}}{\begin{array}{c} 2 \end{array}}-T_{base}$$

where $T_{max}$ is the daily maximum temperature, $\;T_{min}$ is the daily minimum temperature and $T_{base}$ (the base temperature) is the minimum temperature at which growth can occur for a plant species. The base temperature of 5 °C is used for timothy. Daily temperatures were recorded from January 2021 to December 2021 at the Swedish Metrological and Hydrological Institute’s weather station 458, Uppsala, 59°90′ N, 17°59′ E. The accumulated growing degree days was calculated from the start of the meteorological growth that is defined as the first five consecutive days with a daily mean temperature above 5 °C in spring 2021 (Figure S3).

The plants in the greenhouse were harvested when the peduncle turned yellow below at least one head of the plant. However, 14 accessions of timothy and 12 accessions of *P. alpinum* remained in the vegetative stage in the greenhouse and did not form elongated or heading tillers. In addition, 36 accessions of timothy reached only the stem elongation stage. The 26 accessions with only vegetative tillers and the 36 accessions with elongated tillers were harvested at the end of the experiment, about 6 months after the vernalization treatment. All tillers were cut 3 cm above the soil surface. The length of 3 randomly selected vegetative tillers, 3 elongated tillers and 3 tillers with heads were then evaluated and the plant height (PH) was estimated as the average of the 9 tillers. For the plants that did not form elongated and heading tillers, the length of 9 vegetative tillers were measured. In addition, the fresh weight (FW) of all tillers was weighed. The plant material was then dried at 60 °C for 48 h and the dry weight (DW) was measured.

In the field, the plants were harvested when 30–50 percent of the tillers on a plant had reached the booting to heading stage. At harvest, the height of five elongated tillers (2 tall, 1 medium height and 2 short tillers) of each plant was measured from soil surface and PH was estimated as the average of these tillers. The tillers were cut 3 cm above the soil surface, and the FW and DW of the plants were recorded. Plants within the same block were harvested within 3 days and the whole experiment was harvested within 12 days.

### 4.6. Data Analysis

Datasets were analyzed using the software RStudio [35] and JMP ver. 15 SAS Institute Inc., Cary, NC, USA. All traits showed normally distributed residuals and were therefore analyzed with parametric Analysis of Variance (ANOVA) tests. Since the number of accessions differed among the three *Phleum* species, and the number of accessions differed within each group of timothy accessions (wild, semi-wild, landraces, breeding lines and cultivars), an unbalanced nested design was used in the ANOVA to compare species initially. In this model, genotype was nested within accession, and accession was nested within species. Genotypes within accession and species were considered as random effect in the nested model. A normal ANOVA was then used for comparisons of different types of accessions within each species. In addition, the LSMeans Differences Tukey HSD test was performed to study the relationship among groups of accessions within species. Student’s *t*-test was used for pairwise comparisons. Phenotypic diversity within each group of accessions was evaluated by calculating the coefficient of variation (*CV*)

(2) $$CV=\frac{\delta }{\mu }\times 100$$

where δ is the standard deviation and μ is the mean of the trait within the group.

To analyze and display relations among accessions based on all studied growth and developmental traits, we used the multivariate analyses principal component analysis (PCA) and cluster analysis. Moreover, Pearson correlation was used to evaluate and estimate the pattern and level of association between traits. The broad-sense heritability (*H*^2^) was estimated for each trait using the R packages “inti” and “variability” in RStudio

(3) $$H^{2}=\frac{\delta_{g}^{2}}{\delta_{p}^{2}}$$

where, $\delta_{g}^{2}$ is the genotypic variance, $\delta_{p}^{2}$ is the phenotypic variance and $\delta_{p}^{2}=\delta_{g}^{2}+\frac{\delta_{e}^{2}}{r}$ in which *r* is the number of replicates.

## Figures and Tables

**Table 1:** Number of accessions and number of plants planted in the field and the survival of accessions and plants after the first winter for different groups of accessions.

Category	Species	Group	Planted (Number)	Survived (Number)	Survival Rate (%)
Accessions	*P. pratense*	Cultivar	56	56	100.0
		Breeding line	10	10	100.0
		Landrace	55	55	100.0
		Wild	61	61	100.0
		Semi-wild	30	30	100.0
	*P. nodosum*	Cultivar	7	7	100.0
		Wild	7	7	100.0
	*P. alpinum*	Wild	18	12	66.6
Plants	*P. pratense*	Cultivar	823	769	93.4
		Breeding line	151	145	96.0
		Landrace	779	741	95.1
		Wild	928	876	94.4
		Semi-wild	440	415	94.3
	*P. nodosum*	Cultivar	90	86	95.6
		Wild	106	97	91.5
	*P. alpinum*	Wild	29	19	65.5

**Table 2:** Mean of each cluster for each studied trait in *P. pratense, P. nodosum* and *P. alpinum* in the field.

Species	Cluster	Number of Accessions	Trait					
			DTS (GDD)	DTB (GDD)	DTH (GDD)	FW (g per Plant)	DW (g per Plant)	PH (cm)
*P. pratense*	I	19	170.1	239.1	291.9	480.9	196.9	94.0
	II	46	182.1	252.0	302.1	448.2	176.7	85.6
	III	28	190.8	266.0	314.4	442.9	173.7	81.8
	IV	43	176.7	243.1	293.9	364.8	148.4	86.7
	V	43	187.5	255.5	305.7	364.0	142.4	78.7
	VI	33	201.4	270.6	318.5	339.3	133.2	71.1
*P. nodosum*	I	5	173.0	233.9	285.6	309.8	121.5	64.5
	II	8	177.1	239.0	288.2	221.9	87.2	58.7
	III	1	234.1	300.1	340.0	212.3	86.6	44.2
*P. alpinum*	I	5	188.9	232.4	279.5	69.0	25.4	27.0
	II	3	164.2	205.9	268.5	168.3	80.7	46.0
	III	4	187.0	252.7	307.0	386.5	136.9	59.6

**Table 3:** Mean of each cluster for each studied trait in *P. pratense, P. nodosum* and *P. alpinum* in the greenhouse.

Species	Cluster	Number of Accessions	Trait					
			DTS (DAE)	DTB (DAE)	DTH (DAE)	FW (g per Plant)	DW (g per Plant)	PH (cm)
*P. pratense*	I	55	159.4	170.2	183.4	92.6	34.2	103.2
	II	37	169.3	180.9	195.6	70.0	25.1	80.2
	III	12	183.7	224.2	252.3	71.4	24.9	87.6
	IV	17	175.1	196.1	217.1	123.1	46.0	96.6
	V	38	160.2	174.5	184.9	143.9	56.9	125.7
*P. nodosum*	I	3	154.7	177.4	193.2	108.3	46.2	105.2
	II	4	156.7	167.5	179.6	141.5	60.1	128.0
	III	1	161.3	200.0	220.0	100.7	43.8	92.1
	IV	5	161.9	170.0	179.9	65.4	27.4	88.8
*P. alpinum*	I	3	161.2	174.7	187.2	3.1	1.6	44.1
	II	1	173.0	198.0	214.0	33.8	12.8	67.1
	III	1	154.0	163.5	176.5	223.6	87.5	132.0

**Table 4:** Genotypic variance (*V_g_*), environmental variance (*V_e_*), phenotypic variance (*V_p_*) and broad-sense heritability (*H*^2^) of days to stem elongation (DTS), days to booting (DTB), days to heading (DTH), fresh weight (FW), dry weight (DW) and plant height (PH) in the field and in the greenhouse for *P. pratense*.

Trial	Trait	V_g_	V_e_	V_p_	H^2^
Field	DTS	0.9	5.6	2.3	0.40
	DTB	0.6	3.8	1.5	0.37
	DTH	0.5	3.2	1.3	0.37
	FW	2449.5	24,289.1	8521.7	0.29
	DW	401.0	3708.0	1328.0	0.30
	PH	48.7	160.6	88.9	0.55
Greenhouse	DTS	99.2	111.6	127.1	0.78
	DTB	286.4	60.1	301.5	0.95
	DTH	505.0	98.9	529.7	0.95
	FW	745.9	3204.1	1546.9	0.48
	DW	125.2	559.9	265.1	0.47
	PH	315.3	1092.8	588.4	0.54

**Table 5:** Studied traits in individual plants of *P. pratense*, *P. nodosum* and *P. alpinum* in the field and in the greenhouse.

Trait	Abbreviation	Description
Fresh weight	FW	Fresh weight (g) of tillers cut at 3 cm above the soil surface
Dry weight	DW	Dry weight (g) of tillers cut at 3 cm above the soil surface
Plant height	PH	The average length of 5 tillers (cm) in the field and the average length of 9 tillers (cm) in the greenhouse
Days to stem elongation	DTS	Number of days from emergence of coleoptile until the first tiller internode started to elongate, and the inflorescence was palpable at least 1 cm above the stem base in about one-fourth of the total number of tillers
Days to booting	DTB	Number of days from emergence of coleoptile until the tip of the inflorescence was palpable in the flag leaf sheath below the flag leaf base in about one fourth of the total number of tillers
Days to heading	DTH	Number of days from emergence of coleoptile until the head was visible above the flag leaf base in about one-fourth of the total number of tillers

## Data Availability

The data presented in this study are available upon request from the corresponding author.

## Supplementary Materials

The following supporting information can be downloaded at: https://www.mdpi.com/article/10.3390/plants12193494/s1. Figure S1: Correlation between days to heading (DTH) in the field and latitude coordinate of the collection site of wild *P. pratense* accessions. Figure S2: Cloning of *Phleum* genotypes planted in the field and in the greenhouse. Figure S3: Accumulated growing degree days (GDD) calculated from meteorological growth start (defined as the first of five consecutive days with daily mean temperature above 5 °C) for 2021 in Uppsala, Sweden based on the climate data from the Swedish Meteorological and Hydrological Institute, Norrköping, Sweden. Table S1: Unbalanced-nested analysis of variance (ANOVA) for the components block, species, accession and genotype for the studied traits days to stem elongation (DTS), days to booting (DTB), days to heading (DTH), fresh weight (FW), dry weight (DW) and plant height (PH) in the field. DF: degrees of freedom, ns: non-significant, *: *p* < 0.05, **: *p* < 0.01. Table S2: Unbalanced-nested analysis of variance (ANOVA) for the components block, species, accession and genotype for the studied traits days to stem elongation (DTS), days to booting (DTB), days to heading (DTH), fresh weight (FW), dry weight (DW) and plant height (PH) in the greenhouse. DF: degrees of freedom, ns: non-significant, *: *p* < 0.05, **: *p* < 0.01. Table S3: Mean and SD of days to stem elongation (DTS), days to booting (DTB), days to heading (DTH), fresh weight (FW), dry weight (DW) and plant height (PH) in *P. pratense*, *P. nodosum* and *P. alpinum* in the field. GDD: Growing degree days. Mean values that do not share the same letter are significantly different among species according to Tukey HSD, *p* < 0.05. Table S4: Mean and SD of days to stem elongation (DTS), days to booting (DTB), days to heading (DTH), fresh weight (FW), dry weight (DW) and plant height (PH) in *P. pratense*, *P. nodosum* and *P. alpinum* in the greenhouse. DAE: days after coleoptile emergence. Mean values that do not share the same letter are significantly different among species accessions according to Tukey HSD, *p* < 0.05. Table S5: Number of accessions of each group in each cluster in *P. pratense*, *P. nodosum* and *P. alpinum* in the field. Table S6: Number of accessions of each group in each cluster in *P. pratense*, *P. nodosum* and *P. alpinum* in the greenhouse. Table S7: Information of the studied accessions of *P. pratense*, *P. nodosum* and *P. alpinum* from the genebank NordGen, Alnarp, Sweden.
