# Supplementary material for: Phenotypic Diversity in Domesticated and Wild Timothy Grass, and Closely Related Species for Forage Breeding
Source: Plants (Basel). 2023 Oct 7;12(19):3494. doi: 10.3390/plants12193494 (PMC10575225; doi:10.3390/plants12193494)
Supplement: Supplementary file 1 [file plants-12-03494-s001.zip › Supplementary Figures S1-S3.pdf]

Supplementary materials to:

Phenotypic Diversity in Domesticated and Wild Timothy Grass, and Closely Related Species for Forage Breeding

Yousef Rahimi [1,*], Girma Bedada [1], Silvana Moreno [1], Anne-Maj Gustavsson [2], Pär K. Ingvarsson [1] and Anna Westerbergh [1]

[1] Linnean Centre for Plant Biology, Department of Plant Biology, BioCenter, Swedish University of Agricultural Sciences, 750 07 Uppsala, Sweden; girma.bedada@slu.se (G.B.); silvana.moreno@slu.se (S.M.); par.ingvarsson@slu.se (P.K.I.); anna.westerbergh@slu.se (A.W.)

[2] Department of Crop Production Ecology, Swedish University of Agricultural Sciences, 901 83 Umeå, Sweden; anne-maj.gustavsson@slu.se (A.-M.G.)

* Correspondence: yousef.rahimi@slu.se

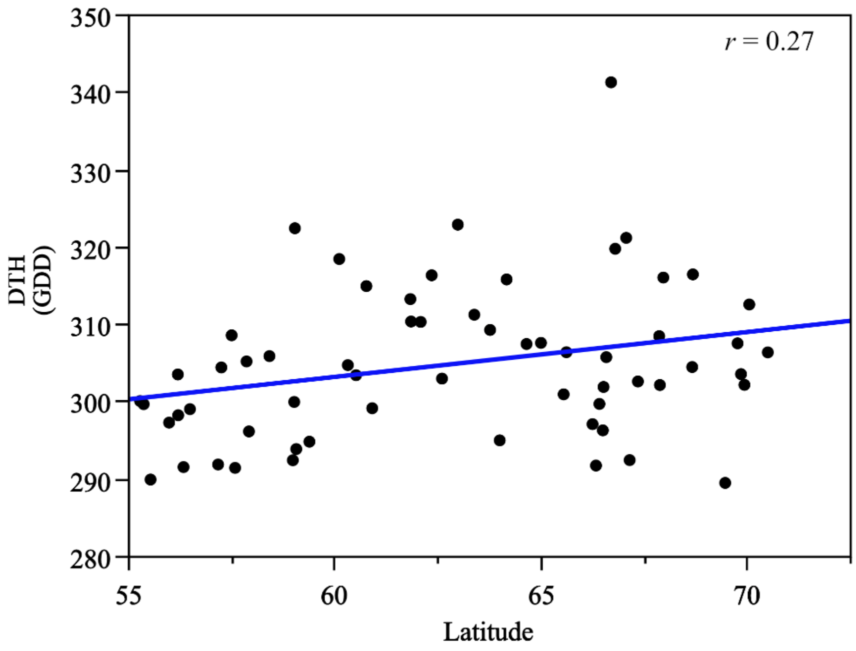

Figure S1. Correlation between days to heading (DTH) in the field and latitude coordinate of the collection site of wild *P. pratense* accessions.

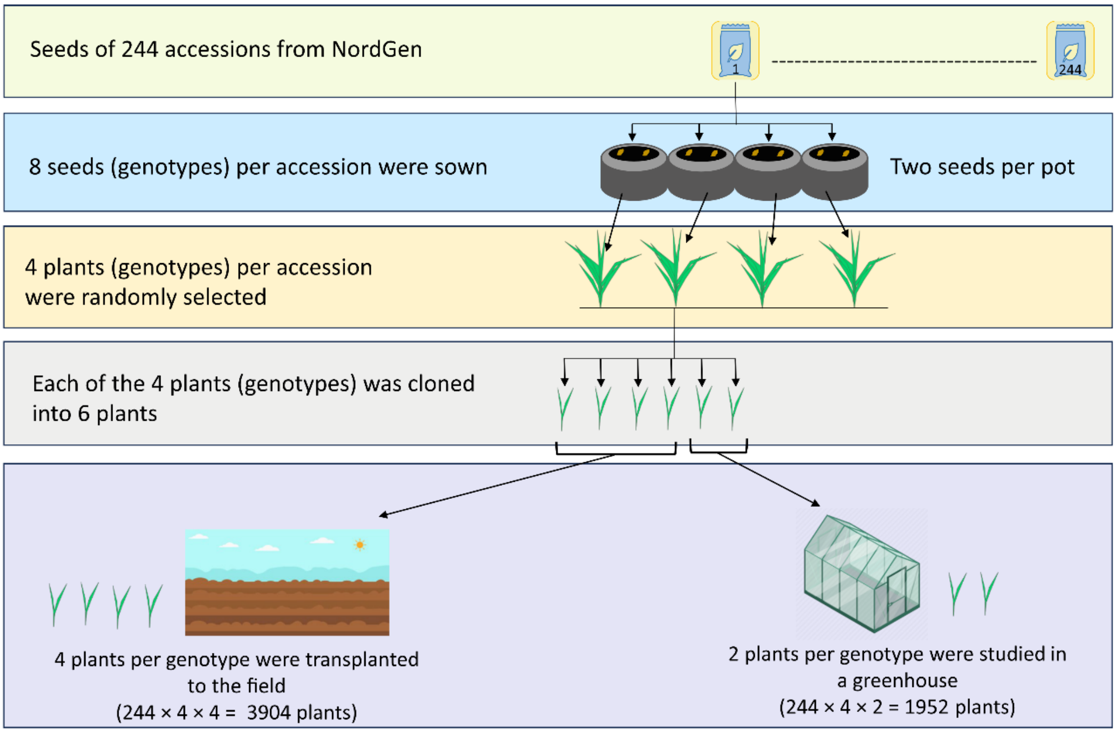

Figure S2. Cloning of *Phleum* genotypes planted in the field and in the greenhouse.

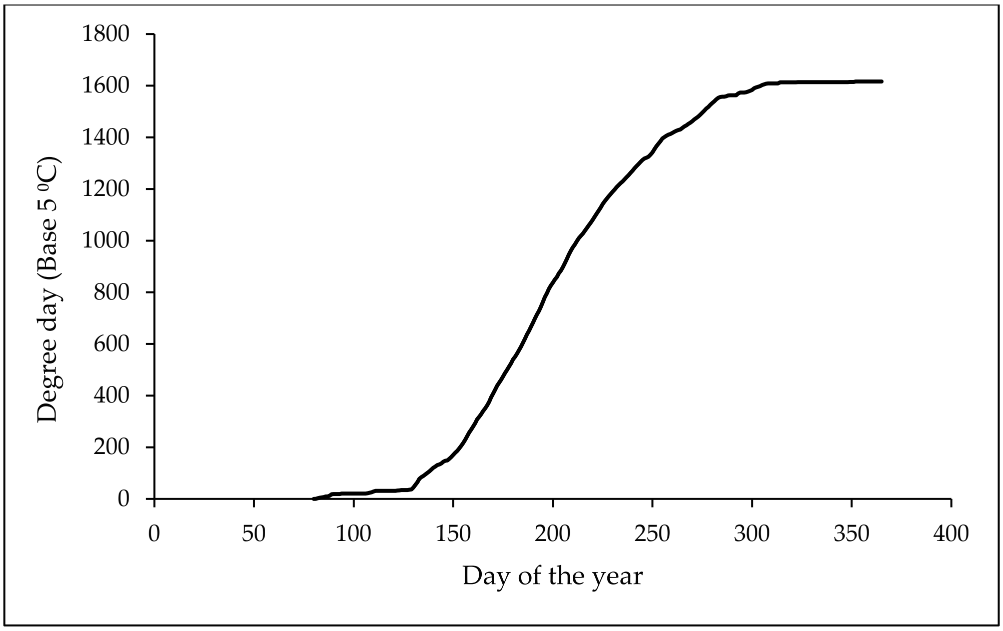

Figure S3. Accumulated growing degree days (GDD) calculated from meteorological growth start (defined as the first of five consecutive days with daily mean temperature above 5 °C) for 2021 in Uppsala, Sweden based on the climate data from the Swedish Meteorological and Hydrological Institute, Norrköping, Sweden.
