# Supplementary material for: Phenotypic Diversity in Domesticated and Wild Timothy Grass, and Closely Related Species for Forage Breeding
Source: Plants (Basel). 2023 Oct 7;12(19):3494. doi: 10.3390/plants12193494 (PMC10575225; doi:10.3390/plants12193494)
Supplement: Supplementary file 1 [file plants-12-03494-s001.zip › Supplementary Tables S1-S6.pdf]

Supplementary materials to:

Phenotypic Diversity in Domesticated and Wild Timothy Grass, and Closely Related Species for Forage Breeding

Yousef Rahimi [1,*], Girma Bedada [1], Silvana Moreno [1], Anne-Maj Gustavsson [2], Pär K. Ingvarsson [1] and Anna Westerbergh [1]

[1] Linnean Centre for Plant Biology, Department of Plant Biology, BioCenter, Swedish University of Agricultural Sciences, 750 07 Uppsala, Sweden; girma.bedada@slu.se (G.B.); silvana.moreno@slu.se (S.M.); par.ingvarsson@slu.se (P.K.I.); anna.westerbergh@slu.se (A.W.)

[2] Department of Crop Production Ecology, Swedish University of Agricultural Sciences, 901 83 Umeå, Sweden; anne-maj.gustavsson@slu.se (A.-M.G.)

* Correspondence: yousef.rahimi@slu.se

Table S1. Unbalanced-nested analysis of variance (ANOVA) for the components block, species, accession and genotype for the studied traits days to stem elongation (DTS), days to booting (DTB), days to heading (DTH), fresh weight (FW), dry weight (DW) and plant height (PH) in the field. DF: degrees of freedom, ns: non-significant, *: $p < 0.05$, **: $p < 0.01$.

Source of Variation	DF	Mean Square					
		DTS	DTB	DTH	FW	DW	PH
Block	3	9566.1**	54169.1**	82409.2**	1033059.0**	399403.0**	25279.9**
Species	2	2429.6*	16319.5**	16604.1**	1764388.0**	277685.0**	44013.9**
Accession (Species)	236	1698.5**	2200.5**	1701.2**	54460.4**	8847.4**	830.7**
Genotype (Accession(Species))&Random	671	930.2	1240.5	957.3	38146.5	5405.4	359.6
RSquare Adj	-	0.4	0.5	0.5	0.3	0.4	0.7

Table S2. Unbalanced-nested analysis of variance (ANOVA) for the components block, species, accession and genotype for the studied traits days to stem elongation (DTS), days to booting (DTB), days to heading (DTH), fresh weight (FW), dry weight (DW) and plant height (PH) in the greenhouse. DF: degrees of freedom, ns: non-significant, *: $p < 0.05$, **: $p < 0.01$.

Source of Variation	DF	Mean Square					
		DTS	DTB	DTH	FW	DM	PH
Block	1	19.2 ^{ns}	17.7 ^{ns}	23.3^{ns}	420.4^{ns}	44.6 ^{ns}	206.9 ^{ns}
Species	2	2297.8**	872.8**	1531.2**	82984.3**	13030.8**	53878.2**
Accession (Species)	210	445.1**	673.0**	1085.0**	7157.8**	1248.3**	2513.2**
Genotype (Accession(Species))&Random	359	153.0	104.8	144.6	4164.1	782.6	1438.4
RSquare Adj	-	0.8	0.9	0.9	0.5	0.5	0.6

Table S3. Mean and SD of days to stem elongation (DTS), days to booting (DTB), days to heading (DTH), fresh weight (FW), dry weight (DW) and plant height (PH) in *P. pratense*, *P. nodosum* and *P. alpinum* in the field. GDD: Growing degree days. Mean values that do not share the same letter are significantly different among species according to Tukey HSD, $p < 0.05$.

**Species**	**DTS (GDD)**	**DTB (GDD)**	**DTH (GDD)**	**FW (g per plant)**	**DW (g per plant)**	**PH (cm)**
P. pratense	185.1±0.5^{ab}	254.4±0.6^{a}	304.4±0.5^{a}	400.2±3.4^{a}	158.9±1.3^{a}	82.4±0.3^{a}
P. nodosum	178.9±2.2^{b}	240.9±2.5^{b}	290.6±2.1^{b}	254.5±14.5^{b}	100.6±5.5^{b}	60.4±1.3^{b}
P. alpinum	187.2±6.5^{a}	237.1±7.1^{b}	291.2±6.4^{b}	186.7±43.6^{b}	72.2±16.4^{b}	44.1±3.9^{c}

Table S4. Mean and SD of days to stem elongation (DTS), days to booting (DTB), days to heading (DTH), fresh weight (FW), dry weight (DW) and plant height (PH) in *P. pratense*, *P. nodosum* and *P. alpinum* in the greenhouse. DAE: days after coleoptile emergence. Mean values that do not share the same letter are significantly different among species accessions according to Tukey HSD, $p < 0.05$.

**Species**	**DTS (DAE)**	**DTB (DAE)**	**DTH (DAE)**	**FW (g per plant)**	**DW (g per plant)**	**PH (cm)**
P. pratense	165.9±0.5^{a}	178.0±0.6^{a}	192.7±0.7^{a}	97.2±2.0^{a}	36.1±0.9^{b}	96.1±1.6^{a}
P. nodosum	158.2±1.4^{b}	170.4±1.6^{b}	182.4±1.9^{b}	103.4±6.8^{a}	43.8±2.9^{a}	105.1±4.0^{a}
P. alpinum	162.9 ±4.8^{ab}	178.0±4.0^{a}	191.0±4.7^{a}	18.8±11.6^{b}	7.5±5.0^{c}	36.2±6.8^{b}

Table S5. Number of accessions of each group in each cluster in *P. pratense, P. nodosum* and *P. alpinum* in the field.

Species	Cluster	Total number of accessions	Number of accessions in each group				
			Cultivar	Breeding line	Landrace	Semi-wild	Wild
P. pratense	I	19	9	-	3	1	6
	II	46	9	2	15	4	16
	III	28	7	1	9	4	7
	IV	43	17	4	6	7	9
	V	43	7	-	11	11	14
	VI	33	7	3	11	3	9
P. nodosum	I	5	3	-	-	-	2
	II	8	4	-	-	-	4
	III	1	-	-	-	-	1
P. alpinum	I	5	-	-	-	-	5
	II	3	-	-	-	-	3
	III	4	-	-	-	-	4

Table S6. Number of accessions of each group in each cluster in *P. pratense, P. nodosum* and *P. alpinum* in the greenhouse.

Species	Cluster	Total number of accessions	Number of accessions in each group				
			Cultivar	Breeding line	Landrace	Semi-wild	Wild
P. pratense	I	55	18	-	18	3	16
	II	37	8	4	9	8	8
	III	12	2	4	-	1	5
	IV	17	1	1	5	2	8
	V	38	20	2	4	2	10
P. nodosum	I	3	3	-	-	-	-
	II	4	1	-	-	-	3
	III	1	1	-	-	-	-
	IV	5	1	-	-	-	4
P. alpinum	I	3	-	-	-	-	3
	II	1	-	-	-	-	1
	III	1	-	-	-	-	1
